# Obesity as a Risk Factor for Failure to Wean from ECMO: A Systematic Review and Meta-Analysis

**DOI:** 10.1155/2021/9967357

**Published:** 2021-05-22

**Authors:** Syed Arsalan A. Zaidi, Kainat Saleem

**Affiliations:** University of Pittsburgh Medical Center, Internal Medicine Department, Pittsburgh, PA, USA

## Abstract

**Purpose:**

Obesity has been associated with an increased risk of respiratory complications and other systemic illnesses. Respiratory dynamics in an obese patient, combined with modified lung physiology of ARDS, present a significant challenge in managing obese patients with ARDS. Many physicians think of obesity as a relative contraindication to ECMO. We performed a meta-analysis to see the effect of obesity on weaning from ECMO and survival to hospital discharge.

**Methods:**

We searched online databases for studies on ECMO and obesity. The search yielded 49 citations in total; after extensive review, six studies were assessed and qualified to be included in the final analysis. Patients were stratified into BMI >30 kg/m^2^ (obese) and BMI < 30 kg/m^2^ (nonobese).

**Results:**

In meta-analysis, there was a total sample population of 1285 patients, with 466 in the obese group and 819 in the nonobese group. There was no significant difference in weaning from ECMO when compared between obese and nonobese patients, with a risk ratio of 1.03 and 95% confidence interval (CI) of 0.94–1.13 (heterogeneity: chi^2^ = 7.44, df = 4 (*p*=0.11), *I*^2^ = 46%). There was no significant difference in survival rates between obese and nonobese patients who were treated with ECMO during hospitalization, with a risk ratio of 1.04 and 95% CI of 0.86–1.25 (heterogeneity: Tau^2^ 0.03, chi^2^ = 14.61, df = 5 (*p*=0.01), *I*^2^ = 66%).

**Conclusion:**

Our findings show no significant difference in survival and weaning from ECMO in obese vs. nonobese patients. ECMO therapy should not be withheld from obese patients, as obesity is not a contraindication to ECMO.

## 1. Introduction

With the rising epidemic of obesity, the significant mortality and morbidity that accompany obesity present a considerable challenge in healthcare [1, 2]. Obesity has been associated as a risk factor for various systemic illnesses such as diabetes, hypertension, hyperlipidemia, obesity hypoventilation syndrome, obstructive sleep apnea, and cardiac diseases, among many others [1, 3, 4]. When considering respiratory dynamics, specific ventilation challenges associated with obese patients include a decrease in total lung capacity (TLC), functional residual capacity (FRC), and vital capacity (VC), as well as increase in pleural pressure and upper and lower airway resistance [5]. Acute respiratory distress syndrome is described by the Berlin definition as an acute diffuse, inflammatory lung injury, leading to increased pulmonary vascular permeability, increased lung weight, and loss of aerated lung tissue, with hypoxemia and bilateral radiographic opacities, associated with increased venous admixture, increased physiological dead space, and decreased lung compliance [6]. Acute respiratory distress syndrome (ARDS) is associated with high mortality rates even without the added challenges of ventilation associated with obesity. Conventional lung-protective ventilation techniques, such as low tidal volume [7, 8], high positive end-expiratory pressures (PEEP) [9], prone positioning [10], and use of neuromuscular blocking agents [11, 12], have shown promise in decreasing morbidity and mortality associated with ARDS. The combination of respiratory dynamics in the overweight population and lung physiology changes in ARDS presents an unprecedented challenge for appropriate management of these patients [5]. Recent studies have shown that patients with SARS-CoV2 and ARDS might be at an even higher risk of mortality [13, 14].

Extracorporeal membrane oxygenation (ECMO) has been used in patients with severe acute respiratory distress syndrome (ARDS) from as early as 1972 as described by Hill et al.[15]. In a randomized controlled trial in 2006 (CESAR [16]), ECMO was shown to be equally safe and effective management of ARDS when compared to conventional techniques of lung-protective ventilation [16]. With technological advancements in extracorporeal ventilation techniques, ECMO use is being seen more often in severe ARDS. ECMO use has been seen as a challenging task in obese patients even with such advanced technologies, primarily due to the difficulty in cannulation and achieving adequate circuit flow [17–20]. Even with successful initial cannulation and circuit flow, obesity is associated with additional risks during ECMO therapy, such as limb ischemia and venous thrombosis [21]. In addition to cannulation and flow issues related to obesity, ECMO therapy in obese adults has also been associated with worse outcomes due to systemic illnesses associated with obesity, such as diabetes mellitus and systemic hypertension [22, 23]. These challenges have led to obesity being recognized as a relative contraindication of ECMO therapy [21, 24, 25].

Multiple recent trials and case reports have looked at ECMO therapy in obese patients. Con et al. reviewed a small sample of 55 patients in 2015 and suggested that obesity is not associated with a higher mortality rate in patients supported by ECMO therapy [26]. Although looked at in multiple small observational studies, no large patient sample exists to see the mortality difference in obese versus nonobese patients supported by ECMO therapy. We performed a systematic review and meta-analysis of multiple observational studies and case series studies that looked at outcomes of ECMO therapy with patients stratified by body mass index (BMI)

## 2. Methods

### 2.1. Study Design

This systematic review was conducted based on Preferred Reporting Items for Systematic reviews and Meta-Analyses (PRISMA) guidelines. The meta-analysis was conducted using REVMAN software.

### 2.2. Eligibility Criteria

We included all randomized controlled trials, observational studies, case series studies, and other retrospective studies which evaluated ECMO therapy and studied the outcomes based on different weight classes, as stratified by body mass index (BMI). All studies between the year 1975 and 2021 were included for further review and screening.

### 2.3. Search Strategy

We searched the databases on MEDLINE, EMBASE, SCOPUS, and the Cochrane Central Register of Controlled Trials (CENTRAL) from January 1975 to January 2021, using a search algorithm developed specifically for this review. Trial registries such as clinicaltrials.gov and International Clinical Trial Registry Platform (ICTRP) were also included in extended search to screen for any potential ongoing or completed trials. The reference lists of identified articles and studies and systematic reviews of ECMO and ARDS were screened for potentially missed articles during the initial search.

### 2.4. Selection and Data Collection

Two authors (SZ and KS) independently evaluated the titles and abstracts of potentially eligible studies. The search had no population restriction. The included studies were published in English, in peer-reviewed journals. Data were extracted independently according to the MOOSE guidelines, and the results were crosschecked. A funnel plot indicated no publication bias in the studies included in the meta-analysis.

### 2.5. Outcome of Interest

The primary outcome of interest for this review was “successful weaning from ECMO” in obese patients (BMI > 30 kg/m^2^) on ECMO therapy as compared to nonobese patients (BMI < 30 kg/m^2^). The secondary outcome of interest was “survival-to-hospital-discharge” in obese patients as compared to nonobese patients. Other outcomes such as all-cause mortality and other outcomes were included in systematic review and discussion where possible, but not in meta-analysis, as these were not provided consistently in all screened studies.

### 2.6. Statistical Analysis

Risk ratios (RRs) were used as the meta-analytic measure of association between obesity and successful weaning from ECMO therapy (study group (obese patients with BMI more than 30 kg/m^2^)) as compared to nonobese patients (control group (patients with BMI less than 30 kg/m^2^)). For each study, the proportion of successfully weaned patients from ECMO therapy was used to calculate RR and corresponding 95% confidence interval (CI) using a 2 × 2 table. The proportion of nonobese patients who were successfully weaned from ECMO therapy was also calculated for each study's control arm.

Heterogeneity between studies was assessed using Cochrane's Q statistic and *I* [1], which is the proportion of total variance observed between the studies attributed to the differences between studies rather than sampling error. *P* value of *Q* statistic less than 0.05 was considered to be significant. RR was also used to analyze the association of obesity with survival to hospital discharge after ECMO therapy between the study group and control group. Funnel plots were created to identify publication bias in the studies included in the meta-analysis. All the analysis for this study was performed using REVMAN software version 5.4.1.

## 3. Results

### 3.1. Description of the Search Results

**Figure 1:**
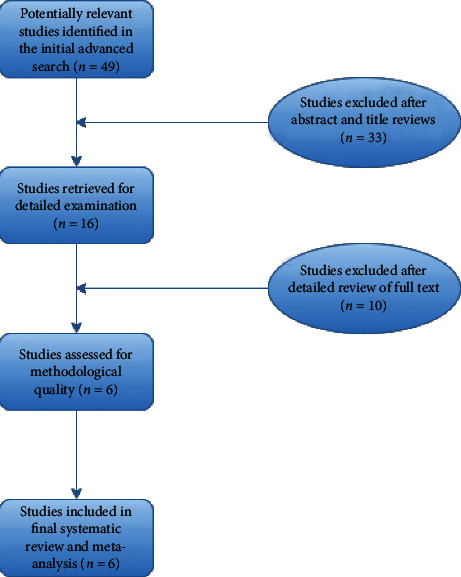
Flowchart for study selection.

The advanced search yielded forty-nine articles in total; these articles' titles and abstracts were then screened for eligibility. Seven duplicates and twenty-six articles were excluded after further review as they did not meet inclusion criteria. The remaining sixteen studies were examined in detail to evaluate the complete reporting of our desired outcome data. Ten of these studies were excluded due to incomplete reporting or data reporting without proper stratification of obesity class in patients. Six remaining studies were assessed for methodological quality, and all six were included in the final meta-analysis (Figure 1).

### 3.2. Description of Studies

The six studies included in the review and meta-analysis were by Cho et al. [27], Dalia et al. [28], Eunmi et al. [29], Galvagno et al. [30], Genore et al. [31], and Salna et al. [32] (Table 1). In all 6 studies, there were no patients excluded from ECMO therapy due to other systemic illnesses, such as diabetes mellitus and chronic hypertension. Cho et al. [27] retrospectively looked at 223 patients from the ARDS registry in Korea and included ARDS patients from 11 hospitals who received ECMO therapy. Cho et al. looked specifically at mortality difference across various weight classes when subjected to ECMO therapy. The study team also reported survival to hospital discharge and successful weaning from ECMO as secondary endpoints, stratified by weight class. The patients were grouped into two classes, less than 25 kg/m^2^ BMI and more than 25 kg/m^2^ BMI. Subgroup analysis was given in the article which was used in our meta-analysis.

Dalia et al. [28] retrospectively studied 355 patients who received ECMO therapy for cardiogenic shock at a single academic center. ECMO therapy outcomes, as well as survival to hospital discharge and weaning from ECMO, were reported as stratified by various weight classes. Eunmi et al. [29] studied outcomes of 200 patients who received ECMO. The article reported outcomes such as survival to hospital discharge, procedural complications, and all-cause mortality in different weight classes. Galvagno et al. [30] retrospectively studied the outcomes of 194 patients on ECMO therapy for respiratory failure at a single center. The study reported outcomes such as death before hospital discharge, ECMO duration, and requirement of additional cannulation. The successful weaning rate was not reported in this study. Thus, this study was not included in the analysis for weaning from ECMO, but included in the analysis for survival to hospital discharge.

Genore et al. [31] retrospectively reviewed charts of 231 patients who received ECMO support. They reported their findings as outcomes of survival to hospital discharge, mortality, length of stay, length of ECMO therapy, weaning from ECMO, and length of ICU stay, as stratified by BMI class. Salna et al. [32] studied the outcomes of 222 patients retrospectively, who were transported for ECMO therapy to their institution from smaller centers and reported the outcomes of survival to discharge and successful weaning, among other outcomes.

### 3.3. Findings and Results

**Figure 2:**
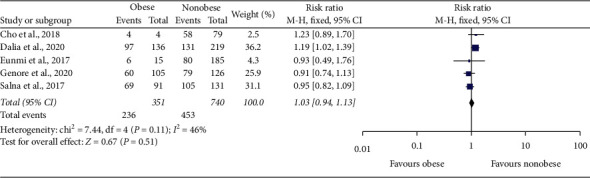
Forest plot of comparison: obese vs. nonobese on ECMO; outcome: successful weaning from ECMO.

**Figure 3:**
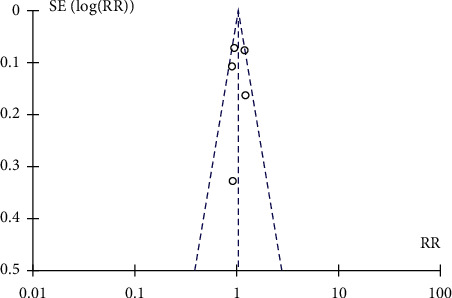
Funnel plot of studies included in the review showing no bias when calculating outcomes of successful weaning from ECMO and its association with obesity.

All studies that were included in this analysis showed that there was no added mortality risk in obese patients when treated with ECMO therapy as compared to nonobese patients. The primary outcome compared in our analysis was “successful weaning from ECMO,” and this outcome was reported in the main article or supplementary material of Cho et al. [27], Dalia et al. [28], Eunmi et al. [29], Genore et al. [31], and Salna et al. [32]. Unfortunately, the article by Galvagno et al. did not report rates of weaning from ECMO, and no supplementary material was available. The 5 studies that were included in the first meta-analysis showed that there was no significant difference in weaning from ECMO when compared between obese and nonobese patients, with a risk ratio of 1.03 and 95% confidence interval (CI) of 0.94–1.13 (heterogeneity: Chi^2^ = 7.44, df = 4 (*p* = 0.11), *I*^2^ = 46%) (Figures 2 and 3 ). All authors agreed that there was no significant association of obesity with failure to wean from ECMO and showed no statistically significant difference in weaning rates when stratified between different subclasses of obesity and normal weight.

**Figure 4:**
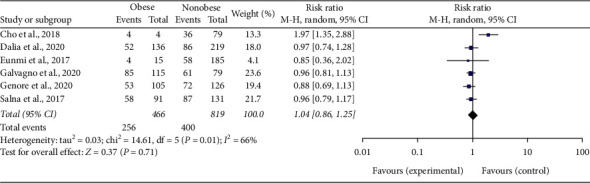
Forest plot of comparison: obese vs. nonobese on ECMO; outcome: survival to hospital discharge.

**Figure 5:**
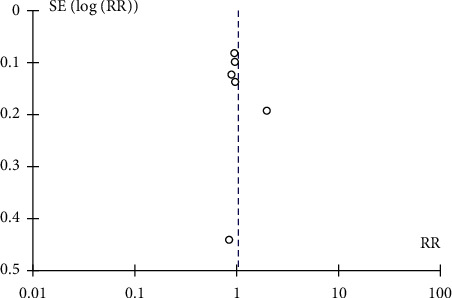
Funnel plot of comparison: obese vs. nonobese on ECMO; outcome: survival to hospital discharge.

All six studies (Cho et al. [27], Dalia et al. [28], Eunmi et al. [29], Galvagno et al. [30], Genore et al. [31], and Salna et al. [32]) were included in the secondary analysis to compare survival to hospital discharge for patients on ECMO and its association with obesity. Since the heterogeneity in this analysis was significant, a random-effect model was used. Our results showed that there was no significant difference in survival rates between obese and nonobese patients who were treated with ECMO during hospitalization; with a risk ratio of 1.04 and 95% confidence interval (CI) of 0.86–1.25 (heterogeneity: Tau^2^ 0.03, Chi^2^ = 14.61, df = 5 (*p*=0.01), *I*^2^ = 66%) (Figure 4). A funnel plot showed no bias between included studies (Figure 5). All authors in included studies concluded that there was no significant difference in survival to hospital discharge (and indirectly mortality rates) between obese and nonobese patients. There was also no significant difference in survival to hospital discharge when these patients were stratified in subclasses of BMI.

## 4. Discussion

Obesity has been associated with an increased risk of developing many systemic illnesses such as diabetes, hypertension, hyperlipidemia, obesity hypoventilation syndrome, obstructive sleep apnea, and cardiac diseases, among many others [1, 3]. Obesity changes respiratory dynamics such as a decrease in total lung capacity (TLC), functional residual capacity (FRC), and vital capacity (VC), as well as increases in pleural pressure and upper and lower airway resistance [5]. When combined with the complicated changes in lung physiology associated with ARDS, this obesity-associated change in respiratory dynamics can be challenging to manage. The use of ECMO has been associated with better survival rates in patients with severe ARDS who have failed conventional therapy [33]. Obesity has often been considered a relative contraindication to ECMO due to difficulty in cannulation and initiating circuit flow due to vascular compression by soft tissue in obese patients. In addition to this, multiple other complications such as the need for second cannulation, limb ischemia, failure to wean from ECMO, and arterial and venous thrombosis have been associated with obesity [20]. Although obesity is not included as a contraindication to ECMO in the guidelines published by Extracorporeal Life Support Organization [25], they mention that individual patient approaches might differ based on physician preference and experience.

Multiple case reports mention the successful use of ECMO in morbidly obese patients [18, 19, 34, 35]. When combined with our analysis of retrospective studies conducted for patients on ECMO with outcomes stratified by weight class, the results of these reports conclude that ECMO should not be withheld from obese patients for fear of failure to wean from ECMO or higher mortality. The studies that were included in our review and analysis were all conducted in a comparable population. All included patients were in intensive care settings or highly specialized ECMO units with a working diagnosis of respiratory failure. Although the studies enrolled patients from all weight classes (BMI 18.5–23 kg/m^2^, BMI 23–24.9 kg/m^2^, BMI 25–29.9 kg/m^2^, BMI 30–39.9 kg/m^2^ (obese), and BMI 40+ kg/m^2^ (morbidly obese)), we stratified the data in two classes: obese (BMI > 30), nonobese (BMI < 30) due to inconsistent reporting of other weight classes in all studies.

Our primary analysis for successful weaning from ECMO in obese vs. nonobese patients was done by analyzing five studies [27–29, 31, 32]. The cumulative risk ratio of successful weaning from ECMO was calculated using REVMAN software and compared between obese and nonobese patients. A total of 1091 patients were included in this analysis, with 351 in the obese group and 740 in the nonobese group. The analysis showed that obese patients were not at a higher risk of failure to wean from ECMO than nonobese patients. Obese patients did not have a statistically significant increased RR of successful weaning from ECMO. These studies' risk ratio was 1.03, with a 95% confidence interval (CI) of 0.94–1.13, which is not clinically or statistically significant as it crosses the value of the null effect (1) and corresponds to a *p* value of 0.11. The studies were consistent with each other based on *I* [1] of 46% which falls within our expected range or 1–50%. Although our patient sample did not explicitly mention patients with BMI more than 45, the use of ECMO in those patients has been separately studied by Kon et al .[26]. Kon et al. studied ECMO therapy outcomes in patients with BMI as high as 66.5 kg/m^2^ and reported better results in patients with class-III obesity (BMI > 45 kg/m^2^). In his review of 55 patients, 100% of patients who had BMI more than 50 kg/m^2^ (*n* = 6) were successfully weaned from ECMO and had zero mortality rate. Galvagno et al. reported 19% mortality in patients with class-III obesity when treated with ECMO, significantly less than the other weight classes in his analysis [30].

Our secondary analysis compared survival to hospital discharge between obese and nonobese patients who received ECMO therapy in the hospital for respiratory failure. This analysis can be interpreted as survival analysis and hospital mortality analysis. A total of 1285 patients were included in this analysis, with 466 in the obese group and 819 in the nonobese group. There was no statistically significant difference when comparing survival to hospital discharge between obese and nonobese patients on ECMO. The risk ratio of survival to hospital discharge was 1.04 with a 95% confidence interval (CI) of 0.86–1.25, which is not clinically significant as it crosses the value of the null effect and but is statistically significant as it corresponds to a *p* value of 0.01. There was heterogeneity in the included studies as evidenced by *I* [1] = 66%, which is slightly above our expected range of 1–50%; thus, a random effect model of analysis was used for these calculations. Our analysis deduced that there was no difference in survival to hospital discharge (or mortality) between obese and nonobese patients treated with ECMO therapy. Thus, ECMO should not be deliberately withheld from obese patients for fear of complications.

Cho et al. [27] suggested a possible beneficial effect of obesity in reducing lung inflammation, which balances out some of the risks associated with changing respiratory dynamics in ARDS. A constant state of low-grade inflammation induced by obesity might play a protective role in ARDS and prevent further insults [36]. Stapleton et al. reported that obesity is associated with lower levels of interleukin-6, interleukin-8, and surfactant protein D, which leads to an altered response to acute lung injury in these patients [37]. Although our analysis did not show any significant difference in mortality between obese and nonobese patients who were treated with ECMO, the findings by Cho et al. and Stapleton et al. are worth noting. Further clinical trials might provide more evidence for any potential mortality benefit of obesity in ECMO.

Keyser et al. [20] also studied ECMO outcomes in 153 obese patients with BMI more than 35 kg/m^2^ and compared them to a control group of 775 patients with BMI < 35 kg/m^2^ and reported that there was no statistical difference in complications between the two groups. His findings also suggested comparable weaning rates and mortality rates between the two groups. Shalaby reviewed 49 patients on ECMO in 2017 and came to similar conclusions [17]. Unfortunately, both articles did not subclassify patients in different weight classes and did not report our desired outcomes in those subgroups; thus, this study was not included in our meta-analysis.

Genore et al. reported that there was no difference in mortality and other significant complications in patients with different weight classes when stratified between venovenous ECMO and venoarterial ECMO [31]. This finding was similar to the conclusion of a much more extensive review of ELSO registry by Kon et al. in 2017, where he reported that venovenous ECMO was not associated with worse outcomes or complications than venoarterial ECMO for ARDS [38]. However, this study population was not stratified by weight class.

In our literature review and systematic analysis, the published data and gathered literature adds to the conclusion that extremes of bodyweight should not be used as an exclusion criterion of ECMO therapy. There might even be some mortality benefits of obesity in ARDS and ECMO therapy [26, 27, 30], though further large randomized clinical trials are needed to support those findings.

### 4.1. Limitations to the Review

Our systematic review has several limitations, including the sample size, the limited number of published studies, especially those in English, and the level of evidence presented in these studies. After an extensive search of all online databases, only 49 articles were identified in the initial phase; many of these studies were excluded because they did not meet our inclusion criteria. Some were excluded because they lacked stratification of patients by BMI and did not report the outcomes we planned to study or did not appropriately define inclusion/exclusion criteria. Although we were successful in achieving a large sample size to conduct our meta-analysis, it is desirable to conduct more head-to-head trials to confirm the significance of our results.

## 5. Conclusion

Obesity has been associated with an increased risk of respiratory complications and other systemic illnesses [4, 39]. The respiratory dynamics in an obese patient, combined with modified lung physiology of ARDS presents a significant challenge in managing obese patients with ARDS. Many healthcare professionals do not consider ECMO therapy for obese patients due to the theoretical risk of increased ECMO complications in this population. Still, our review of multiple recent trials demonstrates that this might be a misconception. There were no significant differences in in-hospital mortality or weaning rates from ECMO compared between obese and nonobese patients. Thus, ECMO should not be withheld in obese patients with severe ARDS who meet ECMO therapy criteria.

## Figures and Tables

**Table 1:** Details of studies included in the analysis and review.

Author	Year	Study design	Single center vs. multicenter	Total number of patients	Stratified by BMI?	Weaning from ECMO numbers reported?	Survival to hospital discharge reported?
Cho et al.	2018	Retrospective	Multicenter	223	Yes	Yes	Yes
Dalia et al.	2020	Retrospective	Single center	355	Yes	Yes	Yes
Eunmi et al.	2017	Retrospective	Single center	200	Yes	Yes	Yes
Galvagno et al.	2020	Retrospective	Single center	194	Yes	No	Yes
Genore et al.	2020	Retrospective	Single center	231	Yes	Yes	Yes
Salna et al.	2017	Retrospective	Single center	222	Yes	Yes	Yes

## Data Availability

The data used to support the findings of this study are available from the corresponding author upon request.

## Abbreviations

ARDS: Acute respiratory distress syndrome
ECMO: Extracorporeal membrane oxygenation
BMI: Body mass index
TLC: Total lung capacity
FRC: Functional residual capacity
VC: Vital capacity
PEEP: Positive end-expiratory pressures
PRISMA: Preferred Reporting Items for Systematic Reviews and Meta-Analyses
MOOSE: Meta-analysis of observational studies in epidemiology
ICU: Intensive care unit
RR: Risk ratio
CI: Confidence interval
SZ: Author, Syed Arsalan Zaidi
KS: Author, Kainat Saleem.
